# Rifaximin Therapy for Patients With Metronidazole-Unresponsive Clostridium difficile Infection

**DOI:** 10.7759/cureus.24140

**Published:** 2022-04-14

**Authors:** Muhammad Waqas, Khadija Mohib, Aniqa Saleem, Mahak LNU, Sabeen Arjumand, Hafiz Habib Ur Rehman Khalil, Rukhshanda Nosheen, Sharmeen Abbas, Kanza M Maqsood, Kiran Abbas

**Affiliations:** 1 Department of Gastroenterology, Lahore General Hospital, Lahore, PAK; 2 Department of Family Medicine, Tarzana Medical Group, Tarzana, USA; 3 Department of Physiology, Sindh Institute of Physical Medicine and Rehabilitation, Karachi, PAK; 4 Department of Medicine, Jinnah Postgraduate Medical Centre, Karachi, PAK; 5 Department of Pharmacology, Sharif Medical and Dental College, Lahore, PAK; 6 Department of Medicine, Lahore General Hospital, Lahore, PAK; 7 Department of Medicine, Services Hospital Lahore, Lahore, PAK; 8 Department of Medicine, People's University of Medical & Health Sciences for Women, Nawabshah, PAK

**Keywords:** rifaximin therapy, metronidazole, metronidazole non responsive, efficacy, clostridium difficile infection

## Abstract

Background

*Clostridium difficile* infection (CDI) is a leading cause of hospital-acquired diarrhea. Rifaximin is an antibiotic that offers marginal resistance to *C. difficile* bacteria. This study was conducted to evaluate the efficacy of rifaximin in metronidazole non-responsive CDI patients.

Methods

A cross-sectional study was performed from August 2019 to May 2020 at the Lahore General Hospital, Lahore, Pakistan. A total of 200 cases were included. Patients who developed diarrhea after receiving antibiotics for seven days and a positive *C. difficile* toxin stool test as detected by the enzyme immunoassay (BioCheck, Inc., CA) were diagnosed with CDI. Only patients who were unresponsive to metronidazole therapy were enrolled in our analysis. Two groups were formed. The intervention group was administered 200 mg tablets of rifaximin three times a day for 10 days. For patients in the control group, no new treatment was started. The efficacy of rifaximin was defined in terms of the resolution of diarrhea after two weeks of therapy and a negative stool test. All data were recorded in a predefined pro forma.

Results

The mean age of 45.41 ± 8.54 years was found in the intervention group. The majority of the patients were aged 35-50 years. The majority of the patients had watery diarrhea, abdominal cramping, and loss of appetite on presentation. Rifaximin was found to be significantly effective in the resolution of symptoms of CDI, which was previously unresponsive to metronidazole (p<0.00001). it was found that the duration of diarrhea of more than three weeks was significantly associated with failure of therapy (p=0.03).

Conclusion

We concluded that rifaximin therapy is effective for patients of CDI non-responsive to metronidazole in more than 65% of the cases. Even though several new developments are made to address the concerned subject, such as microbiota transplantation, antibiotics, and immunotherapy, rifaximin can be considered for patients with metronidazole non-responsive CDI.

## Introduction

*Clostridium difficile* infection (CDI) is primarily a healthcare-associated infection and is a leading cause of hospital-acquired diarrhea. Out of all cases of antibiotic-associated diarrhea, 10%-20% occur due to CDI [1]. The incidence along with the severity of CDI has increased dramatically in the last 10-20 years and it has even affected low-risk populations outside the healthcare system.

Unfortunately, metronidazole therapy has become ineffective in many cases due to an increase in resistant strains [2]. The exact cause of failure is unknown but the possibilities include the emerging resistance of bacteria, increasing disease severity, and poor immune-mediated response in patients. Patients who receive antibiotics get susceptible to CDI as the normal flora of their gut gets compromised. The susceptibility persists for a variable time span from the administration of the last dose of the antibiotic [1,2]. A case-control study suggested that the risk of CDI increased during the usage of antibiotics and even three months after the cessation of antibiotics. The risk is maximum in the first month [3]. The duration depends upon the molecular size of antibiotics, being shorter for cephalosporins and longer for clindamycin. Although, every antibiotic carries the risk of development of CDI, drugs such as clindamycin, cephalosporins, and lately fluoroquinolones carry the highest risk [4].

The mainstay of the treatment lies in the cessation of the causative antibiotic as soon as possible. Treatment with antibiotics, other than those used in the treatment of CDI, leads to the prolongation of diarrhea and recurrent illness. Recurrence is often seen after the cessation of anti-CDI antibiotics. It presents as a reinfection or a new infection. Studies have shown that the rate of recurrence after the initial treatment with oral metronidazole or oral vancomycin is almost 25% [5]. After the first recurrence of infection, the incidence of additional recurrences increases by 40% making the quality of life poor and treatment options limited. To minimize recurrence, the treatment regimen known as pulse therapy is considered using vancomycin for two weeks and then tapering it off for six to seven weeks giving intestinal microbiota the time to restore.

Rifaximin is an antibiotic that offers marginal resistance to *C. difficile* bacteria and is being examined for patients with recurrent CDI [6]. Rifaximin selectively acts on the gut and shows minimum antibiotic resistance when used as prescribed [7]. Clinical results showed that the use of rifaximin for CDI resolved diarrheal symptoms after two weeks in patients who have more than three recurrence episodes [8]. It has been used as a second drug in chaser therapy as it is a nonabsorbable and flora-sparing drug.

This drug has been used to counter the recurrence rate of CDI and the results have been enlightening. Rifaximin has also played a role in alleviating the risk of hepatic encephalopathy in patients with decompensated liver disease and even for Crohn's disease [9,10]. Due to the dearth of local literature, the present study was conducted to determine the efficacy of rifaximin therapy for metronidazole non-responsive patients of CDI.

## Materials and methods

A cross-sectional study was conducted at Lahore General Hospital, Lahore, Pakistan, from August 2019 to May 2020. Participants were recruited using a non-randomized consecutive sampling technique from a public sector hospital. The study was ethically cleared by the institutional review board of Lahore General Hospital (IRB/Gastroenterology-5422).

By using the WHO sample size calculator, keeping the efficacy of 73%, the confidence level of 95%, and the absolute precision of 7%, a sample size of 160 was determined [11]. All individuals with ages ranging from 18 to 70 years, irrespective of gender, diagnosed with CDI unresponsive to metronidazole were eligible to partake in the study. All pregnant patients, lactating mothers, patients with a total leukocyte count of greater than 20,000 cells/mm^3^, renal failure, exposure to vancomycin or rifampin within six weeks before the study were excluded from the study.

All participants were subclassified as those who were administered rifaximin (study group) and those who were continued with metronidazole (control group). All the participants were requested to give informed written consent before data collection. Information collected comprised age, sex, address, contact number, improvement in the resolution of diarrhea after the completion of 14-day therapy of rifaximin. A self-conducted interview technique was employed using a predefined pro forma.

Patients who developed diarrhea after receiving antibiotics for seven days and a positive stool test for *C. difficile* toxin as detected by the enzyme immunoassay (BioCheck, Inc., CA) were diagnosed with CDI. Individuals without the resolution of symptoms of diarrhea after five days of metronidazole therapy were diagnosed with CDI unresponsive to metronidazole. Only patients who were unresponsive to metronidazole therapy were enrolled in our analysis. Two groups were formed. The intervention group was administered 200 mg tablets of rifaximin three times a day for 10 days. For patients in the control group, metronidazole (500 mg, three times a day) was continued.

Participants visited the hospital at four weeks and three months of rifaximin therapy for the identification of CDI and the side effects of the drug. The efficacy of rifaximin was defined in terms of the resolution of diarrhea after two weeks of therapy and a negative stool test.

All of the data were uploaded to IBM SPSS Statistics, version 26 (IBM Corp., Armonk, NY) and analysed appropriately. All quantitative data, including age, were presented using a mean and standard deviation. For all qualitative characteristics such as gender and efficacy, frequency and percentage were determined. The chi-square test was used to determine efficacy. A p-value of less than 0.05 was regarded as significant.

## Results

The mean age of 45.41 ± 8.54 years was found in the intervention group. The majority of the patients were aged 35 to 50 years. The mean age in the study group and the control group was 45.41 ± 8.54 years and 44.5 ± 8.1 years, respectively. In addition to having watery diarrhea on presentation, the majority of the patients in either group also had abdominal cramping as well as loss of appetite. See Table 1 for details.

**Table 1:** Distribution of patient characteristics in the study group versus control group

Characteristics	Rifaximin (n=200)	Control (n=150)	p-value
Age (years)	45.41 ± 8.54	44.5 ± 8.1	0.554
Gender			0.013
Male	104 (52%)	58 (39%)	
Female	96 (48%)	92 (61%)	
Residence			0.006
Urban	148 (74%)	129 (86%)	
Rural	52 (26%)	21 (14%)	
Comorbidities			
Hypertension	86 (43%)	54 (36%)	0.186
Diabetes mellitus type II	108 (54%)	89 (59%)	0.32
Ischemic heart disease	46 (23%)	39 (26%)	0.517
Body mass index	23.4 ± 3.66	24.6 ± 8.3	0.522
Clinical symptoms at presentation			
Diarrhea	200 (100%)	150 (100%)	1
Abdominal cramping	134 (67%)	96 (64%)	0.558
Loss of appetite	118 (59%)	76 (51%)	0.121

Rifaximin was found to be significantly effective in the resolution of symptoms of *C. difficile* that was previously unresponsive to metronidazole (p<0.00001) (Table 2).

**Table 2:** Effectiveness of rifaximin treatment in patients with CDI unresponsive to metronidazole CDI, *Clostridium difficile* infection

Effectiveness	Study group	Control group	p-value
Yes	132 (66%)	55 (36.67%)	<0.00001
No	68 (34%)	95 (63.3%)	
Total	200 (100%)	150 (100%)	

Upon further stratification, it was found that the efficacy of rifaximin therapy did not significantly alter with respect to age or gender. However, it was found that the duration of diarrhea of more than three weeks was significantly associated with failure of therapy (55.3% vs. 70.5%) as illustrated in Table 3.

**Table 3:** Association of effectiveness of rifaximin therapy with the sociodemographic and clinical parameters

Parameters	Efficacy		p-value
	Yes (132)	No (68)	
Age (years)			0.36
35-50	84 (63.6%)	48 (70.6%)	
51-70	48 (36.4%)	20 (29.4%)	
Gender			0.41
Male	65 (49.2%)	38 (55.9%)	
Female	67 (50.8%)	30 (44.1%)	
Duration of diarrhea			
1-3 weeks	59 (44.7%)	20 (29.4%)	0.036
>3 weeks	73 (55.3%)	48 (70.5%)	

## Discussion

Rifaximin was found to be significantly effective in the resolution of symptoms of CDI, which was previously unresponsive to metronidazole. Furthermore, it was found that the duration of diarrhea of more than three weeks was significantly associated with the failure of therapy.

The present study coincides with a previously published study carried out by Basu et al. that elucidated that 73% of CDI cases non-responsive to metronidazole responded to rifaximin and eradication occurred after two weeks [11]. Various case reports indicated that rifaximin has an effect against CDI [12,13]. A few randomized studies have shown that rifaximin when used after the standardized therapy with conventional antibiotics, i.e. metronidazole and vancomycin, had a much better outcome than placebo [14].

In the study carried out by Mattila et al., in 2012, the observed results were much promising as rifaximin cured 53% of patients of recurrent CDI [6]. Alongside the positive cure rate, rifaximin did not induce any side effects in these patients. Moreover, the treatment in all patients was preceded by metronidazole and vancomycin, but rifaximin contributed the maximum to the treatment. In another series of experiments, almost 85% of patients having CDI recovered after the first course of rifaximin, and others who had recurrences had no further diarrhea after the second course of rifaximin therapy [15].

A placebo-controlled trial study used rifaximin immediately after microbial therapy for CDI and approximately 80% of patients responded favorably to the treatment [16]. Taken together, the cure rate of the above-mentioned studies is evidently higher than the cure rate of 65% in our study. One possible explanation is our patients were more prone to relapses that might have contributed to the decreased outcome as compared with the results observed in past studies. The predictable factors influencing the recovery rate are increased age, low albumin levels, disease severity, exposure to the hospital environment, and use of proton pump inhibitors [17-22].

Marchese et al. in their study discussed the possibility of the therapeutic use of rifaximin in gastrointestinal (GIT) diseases, cirrhosis, and possible portal encephalopathy [23]. Furthermore, low-resistant populations showed that high levels of the drug that reached the GIT tract were helpful in preventing mutations. Garey et al. concluded that giving rifaximin 1200 mg for two weeks continued by two weeks of half the dose was effective in resolving *C. difficile*-associated diarrhea and avoiding recurrence in the majority of patients having a response rate of 83% [24].

Similarly, Ng et al. reported rifaximin being an effective alternative therapy for the management of *C. difficile *infections thereby reducing the recurrence of infections as it only gets minimally absorbed in systemic circulation [25]. However, the authors also discussed clinical studies in which resistance to the drug had a major effect on its effectiveness (29.1%-48.9%) against the infection. Moreover, in a pilot study by Garey et al., individuals with CDI who were prescribed rifaximin were found to have a reduced rate of recurrent diarrhea (21%) (p=0.018) in contrast to placebo (49%) whereas recurrence of CDI was higher in the placebo group (31%) as compared to the rifaximin group (15%) (p=0.11) [13].

Berman found recurrence of *C. difficile*-associated diarrhea to resolve with a combination of drugs such as probiotics, vancomycin, and rifaximin for a duration of seven weeks [12]. In a different study by Tannous et al., rifaximin was also seen to be beneficial for managing the recurrence of CDI as no recurrence was observed even after six months of treatment had ended [26].

In our study, we found some limitations. For instance, we tried to analyse if the association of these above-mentioned factors with rifaximin could be assessed, but the sample turned out to be small to address this. Furthermore, due to the small sample size, the findings of the study cannot be generalized to a larger cohort.

Nevertheless, the development of resistance against rifaximin is a potential concern. This is attributed to genetic mutations in the beta subunit of ribonucleic acid (RNA) polymerase [27]. However, extensive research is required to explore the factors associated with rifaximin resistance to develop strategies to mitigate the process.

## Conclusions

In conclusion, rifaximin treatment was found to be significantly effective in the resolution of symptoms of CDI unresponsive to metronidazole therapy. Moreover, we also found that the duration of diarrhea of more than three weeks was significantly associated with the failure of therapy. Further large-scale studies are required to confirm the claim that rifaximin is effective in completely curing the Clostridium infection resistant to conventional therapy.
